# Supplementary material for: Evaluating the transcriptional regulators of arterial gene expression via a catalogue of characterized arterial enhancers
Source: eLife. 2025 Jan 17;14:e102440. doi: 10.7554/eLife.102440 (PMC11896612; doi:10.7554/eLife.102440)
Supplement: Table 2—source data 1. — ‘Selected’ indicates that the region meets our threshold as a putative enhancer, ‘exception’ indicates region did not meet our threshold but was included in transgenic analysis as a control (grey text). Numbers indicate approximate distance from the TSS of the named arterial gene. * indicates that enhancer mark was widely seen beyond endothelial cells. Grey italic text refers to regions previously implicated in enhancer activity, with the /enhancer name ascribed in the original reference. Cxcr4-117/CXCR4-125 is from Tsaryk et al., 2022 ; Cxcr4-1, Nrp1- 1/NRP1A and Nrp1+76/NRP1B are from Yamamizu et al., 2010 ; Efnb2+17/EFNB2A and Efnb2+25/EFNB2B are from Grego-Bessa et al., 2007; and Efnb2+4/EFNB2R1 and Efnb2+28/EFNB2R4 are from Stewen et al., 2024. [file elife-102440-table2-data1.docx]

**Table 2 – Source data 1**

Enhancer marks in different human and mouse ECs at putative enhancer regions within the loci of eight arterial genes. “Selected” indicates that the region meets our threshold as a putative enhancer, “exception” indicates region did not meet our threshold but was included in transgenic analysis as a control (grey text). Numbers indicate approximate distance from the TSS of the named arterial gene. * indicates that enhancer mark was widely seen beyond endothelial cells. Grey italic text refers to regions previously implicated in enhancer activity, with the /enhancer name ascribed in the original reference. *Cxcr4-117/CXCR4-125* is from ^68^; *Cxcr4-1*, *Nrp1-1/NRP1A* and *Nrp1+76/NRP1B* are from ^70^; *Efnb2+17/EFNB2A* and *Efnb2+25/EFNB2B* are from ^66^; and *Efnb2+4/EFNB2R1* and *Efnb2+28/EFNB2R4* are from ^69^.

| **Enhancer** | **H DNAseI** | **H histone** | **M artery ATAC** | **M retina ATAC** | **M E11 p300** | **Selected** | **Exception** |
| --- | --- | --- | --- | --- | --- | --- | --- |
| ***Acvrl1-5*** | YES | YES | NO | NO | YES | **SELECTED** |  |
| ***Acvrl1-1/p*** | YES | YES | NO | YES | YES | **SELECTED** |  |
| ***Acvrl1+6*** | YES | YES | NO | YES | YES | **SELECTED** |  |
| *Acvrl1+16* | NO | NO | YES | YES | YES | NO | MOUSE ONLY |
| ***Acvrl1+19*** | YES | YES | YES | NO | YES | **SELECTED** |  |
| *Cxcl12-184* | YES | YES | NO | NO | NO | NO | HUMAN ONLY |
| ***Cxcl12-2*** | NO | NO | YES | YES | NO | **SELECTED** |  |
| *Cxcl12+239* | NO | NO | NO | YES | NO | NO | 1 MARK ONLY |
| *Cxcl12+265* | NO | NO | NO | YES | NO | NO | 1 MARK ONLY |
| ***Cxcl12+269*** | NO | NO | YES | YES | NO | **SELECTED** |  |
| ***Cxcl12+298*** | YES | YES | NO | YES | YES | **SELECTED** |  |
| ***Cxcl12+376*** | YES | NO | YES | NO | NO | **SELECTED** |  |
| ***Cxcl12+383*** | NO | YES | YES | YES | YES | **SELECTED** |  |
| *Cxcl12+439* | YES | YES | NO | NO | NO | NO | HUMAN ONLY |
| ***Cxcl12+445*** | YES* | YES | YES | YES | NO | **SELECTED** |  |
| ***Cxcr4-232*** | YES | NO | NO | YES | NO | **SELECTED** |  |
| ***Cxcr4-194*** | NO | NO | YES | NO | YES | **SELECTED** |  |
| ***Cxcr4-130*** | NO | NO | NO | YES | YES | **SELECTED** |  |
| *Cxcr4-117/CXCR4-125* | YES | YES | NO | NO | NO | NO | LITERATURE |
| ***Cxcr4-113*** | YES | YES | YES | YES | YES | **SELECTED** |  |
| ***Cxcr4-109*** | YES | YES | YES | YES | YES | **SELECTED** |  |
| ***Cxcr4+1*** | NO | NO | YES | YES | NO | **SELECTED** |  |
| ***Cxc4+119*** | NO | YES | YES | YES | YES | **SELECTED** |  |
| ***Cxcr4+135*** | YES | YES | YES | YES | YES | **SELECTED** |  |
| ***Cxcr4+151*** | YES | YES | NO | YES | YES | **SELECTED** |  |
| ***Efnb2-333*** | YES | YES | YES | YES | YES | **SELECTED** |  |
| ***Efnb3-159*** | YES | YES | NO | YES | YES | **SELECTED** |  |
| ***Efnb2-141*** | YES | YES | YES | YES | YES | **SELECTED** |  |
| ***Efnb2-112*** | YES | YES | NO | YES | YES | **SELECTED** |  |
| ***Efnb2+3*** | YES* | YES | YES | NO | NO | **SELECTED** |  |
| *Efnb2+4/EFNB2 R1* | YES | NO | NO | NO | NO | NO |  |
| *Efnb2+17/EFNB2 A* | NO | YES | NO | NO | NO | NO |  |
| *Efnb2+25/EFNB2 B* | NO | NO | NO | NO | NO | NO |  |
| *Efnb2+28/EFNB2 R4* | NO | NO | NO | NO | NO | NO |  |
| ***Efnb2+37*** | YES | YES | YES | YES | YES | **SELECTED** |  |
| ***Efnb2+172*** | YES | YES | YES | NO | YES | **SELECTED** |  |
| ***Efnb2+209*** | YES* | YES | NO | YES | YES | **SELECTED** |  |
| ***Gja+24*** | NO | YES | YES | NO | YES | **SELECTED** |  |
| ***Gja4+50*** | YES | NO | YES | YES | YES | **SELECTED** |  |
| *Gja4+57* | NO | NO | NO | NO | YES | NO | MOUSE ONLY |
| ***Gja5-93*** | NO | NO | YES | YES | YES | **SELECTED** |  |
| ***Gja5-78*** | NO | YES | YES | YES | YES | **SELECTED** |  |
| ***Gja5-28*** | YES | NO | YES | NO | NO | **SELECTED** |  |
| *Gja5-21* | YES | YES | NO | NO | NO | NO | HUMAN ONLY |
| ***Gja5-7*** | YES | YES | YES | YES | NO | **SELECTED** |  |
| *Nrp1-1/NRP1 A* | YES* | NO | NO | NO | NO | NO |  |
| ***Nrp1+28*** | NO | YES | NO | YES | YES | **SELECTED** |  |
| *Nrp1+76/ NRP1 B* | YES* | YES | NO | NO | NO | NO | LITERATURE |
| ***Nrp1+78*** | YES | YES | NO | YES | YES | **SELECTED** |  |
| ***Nrp1+91*** | YES | YES | YES | YES | YES | **SELECTED** |  |
| ***Nrp1+129*** | YES | YES | NO | YES | YES | **SELECTED** |  |
| ***Unc5b-57*** | NO | YES | NO | NO | YES | **SELECTED** |  |
| *Unc5b+14* | NO | NO | YES | YES | NO | NO | MOUSE ONLY |
| ***Unc5b+23*** | YES* | YES | YES | YES | YES | **SELECTED** |  |
| ***Unc5b+30*** | YES | YES | NO | YES | YES | **SELECTED** |  |
| ***Unc5b+39*** | YES | YES | NO | YES | YES | **SELECTED** |  |
| *Unc5b+43* | YES | YES | NO | NO | NO | NO | HUMAN ONLY |
