# Supplementary material for: Evaluating the transcriptional regulators of arterial gene expression via a catalogue of characterized arterial enhancers
Source: eLife. 2025 Jan 17;14:e102440. doi: 10.7554/eLife.102440 (PMC11896612; doi:10.7554/eLife.102440)
Supplement: Table 2—source data 2. — Values given for mm10 represent the core enhancer regions analysed for motif sequences and protein binding. [file elife-102440-table2-data2.docx]

**Table 2 –Source data 2**

Genome locations for all enhancer (and human orthologues) investigated in this paper. Values given for mm10 represent the core enhancer regions analysed for motif sequences and protein binding.

| **ARTERIAL ENHANCERS** | mm10 of CORE | mm9 | hg38 | hg19 |
| --- | --- | --- | --- | --- |
| ***Cxcr4-194*** | chr1:128,785,634-128,786,023 | chr1:130,681,776-130,682,867 | chr2:136,325,218-136,326,027 | chr2:137,082,788-137,083,597 |
| ***Cxcr4+135*** | chr1:128,457,037-128,457,301 | chr1:130,353,525-130,353,952 | chr2:136,014,017-136,014,436 | chr2:136,771,587-136,772,006 |
| ***Cxcr4+151*** | chr1:128,440,683-128,440,929 | chr1:130,337,167-130,337,580 | chr2:136,000,071-136,000,490 | chr2:136,757,641-136,758,060 |
| ***Efnb2-333*** | chr8:8,994,388-8,994,688 | chr8:8,994,342-8,994,890 | chr13:106,919,439-106,920,184 | chr13:107,571,787-107,572,532 |
| ***Efnb2-159*** | chr8:8,819,393-8,819,702 | chr8:8,819,393-8,819,702 | chr13:106,701,743-106,702,339 | chr13:107,354,284-107,354,541 |
| ***Efnb2-141*** | chr8:8,801,731-8,802,062 | chr8:8,801,433-8,802,174 | chr13:106,680,416-106,681,557 | chr13:107,332,764-107,333,905 |
| ***Efnb2-112*** | chr8:8,772,250-8,772,661 | chr8:8,772,171-8,772,912 | chr13:106,651,110-106,652,084 | chr13:107,303,458-107,304,432 |
| ***Gja4+50*** | chr4:127,263,807-127,264,170 | chr4:126,940,851-126,941,567 | chr1:34,842,968-34,843,574 | chr1:35,308,569-35,309,175 |
| ***Unc5b+30*** | chr10:60,807,701-60,807,963 | chr10:60,263,361-60,264,141 | chr10:71,254,099-71,255,108 | chr10:73,013,856-73,014,865 |
| ***Unc5b+39*** | chr10:60,800,879-60,801,144 | chr10:60,255,453-60,256,125 | chr10:71,263,111-71,264,120 | chr10:73,022,868-73,023,877 |
| ***Acvrl1+6*** | chr15:101,134,090-101,134,328 | chr15:100,964,449-100,964,836 | chr12:51,911,761-51,912,189 | chr12:52,305,545-52,305,973 |
| ***Cxcl12+269*** | chr6:117,437,703-117,437,974 | chr6:117,387,534-117,388,459 | chr10:44,043,453-44,044,439 | chr10:44,538,901-44,539,887 |
| ***Gja5-78*** | chr3:96,953,907-96,954,116 | chr3:96,757,582-96,758,245 | chr1:147,877,925-147,878,475 | chr1:147,349,718-147,350,707 |
| ***Gja5-7*** | chr3:97,025,457-97,025,703 | chr3:96,829,228-96,829,714 | chr1:147,781,009-147,781,500 | chr1:147,253,120-147,253,611 |
| ***Nrp1+78*** | chr8:128,437,372-128,437,681 | chr8:130,961,192-130,961,715 | chr10:33,249,630-33,250,192 | chr10:33,538,558-33,539,120 |
| *DLL4-12** | chr2:119,314,902-119,315,163 | chr2:119,140,274-119,141,353 | chr15:40,918,508-40,919,627 | chr15:41,210,706-41,211,825 |
| *DLL4in3** | chr2:119,327,292-119,327,550 | chr2:119,152,838-119,153,684 | chr15:40,930,683-40,931,372 | chr15:41,222,881-41,223,570 |
| *ECE1** | chr4:137,920,171-137,920,428 | chr4:137,475,719-137,476,738 | chr1:21,279,545-21,280,564 | chr1:21,606,038-21,607,057 |
| *Flk1in10** | chr5:75,962,034-75,962,270 | chr5:76,357,891-76,358,715 | chr4:55,106,811-55,107,736 | chr4:55,972,978-55,973,903 |
| *Hey1-18** | chr3:8,685,313-8,685,498 | chr3:8,685,099-8,685,821 | chr8:79,783,375-79,784,874 | chr8:80,695,610-80,697,109 |
| *NOTCH1+16** | chr2:26,490,736-26,491,004 | chr2:26,346,100-26,346,671 | chr9:136,530,091-136,530,501 | chr9:139,424,543-139,424,953 |
| *Sema6d-55** | chr2:124,555,118-124,555,549 | chr2:124,380,522-124,381,285 | chr15:47,665,826-47,666,567 | chr15:47,958,023-47,958,764 |
| *SOX7+14** | chr14:63,957,836-63,958,080 | chr14:64,576,271-64,577,533 | chr8:10,715,575-10,716,781 | chr8:10,573,085-10,574,291 |
| *Unc5b-57 (appears venous)* | chr10:60,888,540-60,888,832 | chr10:60,351,050-60,351,950 | chr10:71,132,227-71,133,212 | chr10:72,891,984-72,892,969 |
| **PAN-EC ENHANCERS** | mm10 of CORE | mm9 | hg38 | hg19 |
| *Apln+28* | chrX:48,006,129-48,006,455 | chrX:45,359,306-45,359,632 | chrX:129,622,779-129,623,183 | chrX:128,756,756-128,757,160 |
| *Cdh5-1* | chr8:104,101,460-104,101,691 | chr8:106,625,360-106,625,591 | chr16:66,366,342-66,366,812 | chr16:66,400,303-66,400,656 |
| *Egfl7-2* | chr2:26,578,567-26,578,781 | chr2:26,434,087-26,434,301 | chr9:136,655,840-136,656,439 | chr9:139,550,292-139,550,891 |
| *Egfl7-9* | chr2:26,571,993-26,572,187 | chr2:26,427,513-26,427,707 | chr9:136,646,298-136,646,847 | chr9:139,540,750-139,541,299 |
| *Eng-8* | chr2:32,638,086-32,638,303 | chr2:32,493,606-32,493,823 | chr9:127,862,259-127,862,525 | chr9:130,624,538-130,624,804 |
| *Fli1+12* | chr9:32,529,710-32,529,953 | chr9:32,337,295-32,337,538 | chr11:128,705,389-128,705,988 | chr11:128,575,436-128,575,782 |
| *Flk1+3* | chr5:75,974,602-75,975,031 | chr5:76,370,627-76,371,056 | chr4:55,121,178-55,121,753 | chr4:55,987,345-55,987,920 |
| *Gata2+9* | chr6:88,203,083-88,203,392 | chr6:88,153,077-88,153,386 | chr3:128,483,128-128,483,430 | chr3:128,201,971-128,202,273 |
| *Mef2cF7* | chr13:83,711,228-83,711,450 | chr13:83,572,070-83,572,292 | chr5:88,827,268-88,827,485 | chr5:88,123,031-88,123,357 |
| *Notch1+33* | chr2:26,475,039-26,475,265 | chr2:26,330,559-26,330,785 | chr9:136,511,904-136,512,203 | chr9:139,406,356-139,406,655 |
| *Pdgfrb+18* | chr18:61,059,590-61,059,912 | chr18:61,219,244-61,219,566 | chr5:150,137,320-150,137,793 | chr5:149,516,883-149,517,356 |
| *Tal1-4* | chr4:115,052,638-115,052,947 | chr4:114,725,243-114,725,552 | chr1:47,235,413-47,235,675 | chr1:47,701,050-47,701,347 |
| *Tie1-1* | chr4:118,489,769-118,490,150 | chr4:118,162,374-118,162,755 | chr1:43,300,547-43,301,064 | chr1:43,766,218-43,766,735 |
| **VEIN ENHANCERS** | mm10 of CORE | mm9 | hg38 | hg19 |
| *CoupTFII-965* | chr7:71,311,521-71,311,881 | chr7:78,456,407-78,456,767 | chr15:95,365,479-95,366,011 | chr15:95,908,708-95,909,240 |
| *Ephb4-2* | chr5:137,348,682-137,349,353 | chr5:137,789,910-137,790,581 | chr7:100,828,715-100,829,637 | chr7:100,426,337-100,427,259 |
| *Mef2cF10* | chr13:83,582,603-83,582,899 | chr13:83,721,761-83,722,057 | chr5:88,815,163-88,815,436 | chr5:88,110,980-88,111,253 |
